# Surgical Techniques to Optimize Early Urinary Continence Recovery Post Robot Assisted Radical Prostatectomy for Prostate Cancer

**DOI:** 10.1007/s11934-017-0717-4

**Published:** 2017-07-17

**Authors:** Ashwin N. Sridhar, Mohammed Abozaid, Prabhakar Rajan, Prasanna Sooriakumaran, Greg Shaw, Senthil Nathan, John D. Kelly, Tim P Briggs

**Affiliations:** 1 Department of Urology, University College London Hospital, London, UK; 2 Division of Surgery and Cancer, University College London, London, UK; 3 Nuffield Department of Surgical Sciences, University of Oxford, Oxford, UK

**Keywords:** Urosurgery, Surgical techniques, Urinary continence, Robotic prostatectomy

## Abstract

**Purpose of Review:**

A variety of different surgical techniques are thought to impact on urinary continence (UC) recovery in patients undergoing robot assisted radical prostatectomy (RARP) for prostate cancer. Herein, we review current evidence and propose a composite evidence-based technique to optimize UC recovery after RARP.

**Recent Findings:**

A literature search on studies reporting on surgical techniques to improve early continence recovery post robotic prostatectomy was conducted on PubMed and EMBASE. The available data from studies ranging from randomized control trials to retrospective cohort studies suggest that minimizing damage to the internal and external urinary sphincters and their neural supply, maximal sparing of urethral length, creating a secure vesicourethral anastomosis, and providing anterior and posterior myo- fascio-ligamentous support to the anastomosis can improve early UC recovery post RARP.

**Summary:**

A composite evidence-based surgical technique incorporating the above principles could optimize early UC recovery post RARP. Evidence from randomized studies is required to prove benefit.

## Introduction

Prostate cancer is the commonest cancer affecting Western men [1]. Over 90% of cases are organ confined at diagnosis [2], and surgery by robot assisted radical prostatectomy (RARP) is now the gold standard extirpative treatment. A significant toxicity of RARP is urinary incontinence, which impacts on patients’ quality of life (QoL) and psychological wellbeing, regardless of oncologic and sexual function outcomes [3, 4].

More than 80% of men will regain urinary continence (using a no pad definition) at 1 year [5], and more will regain up to 2 years after the operation [6]. Early urinary continence (UC) rates however are much worse. In fact, the likelihood of a patient requiring pads after surgery is typically 70–80% at 6 weeks, 50–60% at 3 months, and 20–40% at 6 months, even among the most experienced surgeons [7].

Multiple factors have been reported to influence recovery of UC post RARP. These include patient factors (age, body mass index, comorbidity, lower urinary tract symptoms, and prostate volume) [8], surgeon experience, and surgical technique. Surgical technique is the only modifiable factor among these, and therefore, identifying and developing an optimal operative technique is likely to impact on continence outcomes [9].

Here, we review the available literature on the surgical technique in UC recovery after RARP and describe an optimal technique that we believe encompasses the different key surgical steps that known to impact on UC.

## Pathophysiology of Urinary Incontinence Post RARP

RARP causes both anatomical and functional alterations in the sphincteric mechanism (internal and external) and surrounding supporting structures in the pelvis (pubourethral ligaments, arcus tendineus fascia, endopelvic fasciae, Denonvilliers’ fascia, and detrusor slips) causing urinary incontinence [10].

In a normal male, the sphincteric mechanism is composed of the internal sphincter (bladder neck) proximally, the external sphincter distally and the connecting longitudinal smooth muscle of the urethra and prostate. The internal sphincter is ring-shaped and made up of smooth muscle fibers from the bladder trigone surrounding the urethra circumferentially, supplied by hypogastric nerves [10, 11]. It is responsible for closure of the bladder—contributing to passive urinary continence and avoiding retrograde ejaculation. Distally, the horseshoe-shaped external sphincter envelopes the membranous urethra, which is deficient posteriorly and expanded anteriorly [10]. The external sphincter comprises an outer layer of striated muscle (also known as the rhabdosphincter), which is under somatic control via the pudendal nerve and an inner layer of smooth muscle, which is involuntary and supplied by the inferior hypogastric plexus (continuous with supply to the membranous urethral smooth muscle). The external sphincter is thought to be responsible for active UC. Injury to either/both sphincters and their neural supply can occur during surgery.

**Fig. 1:**
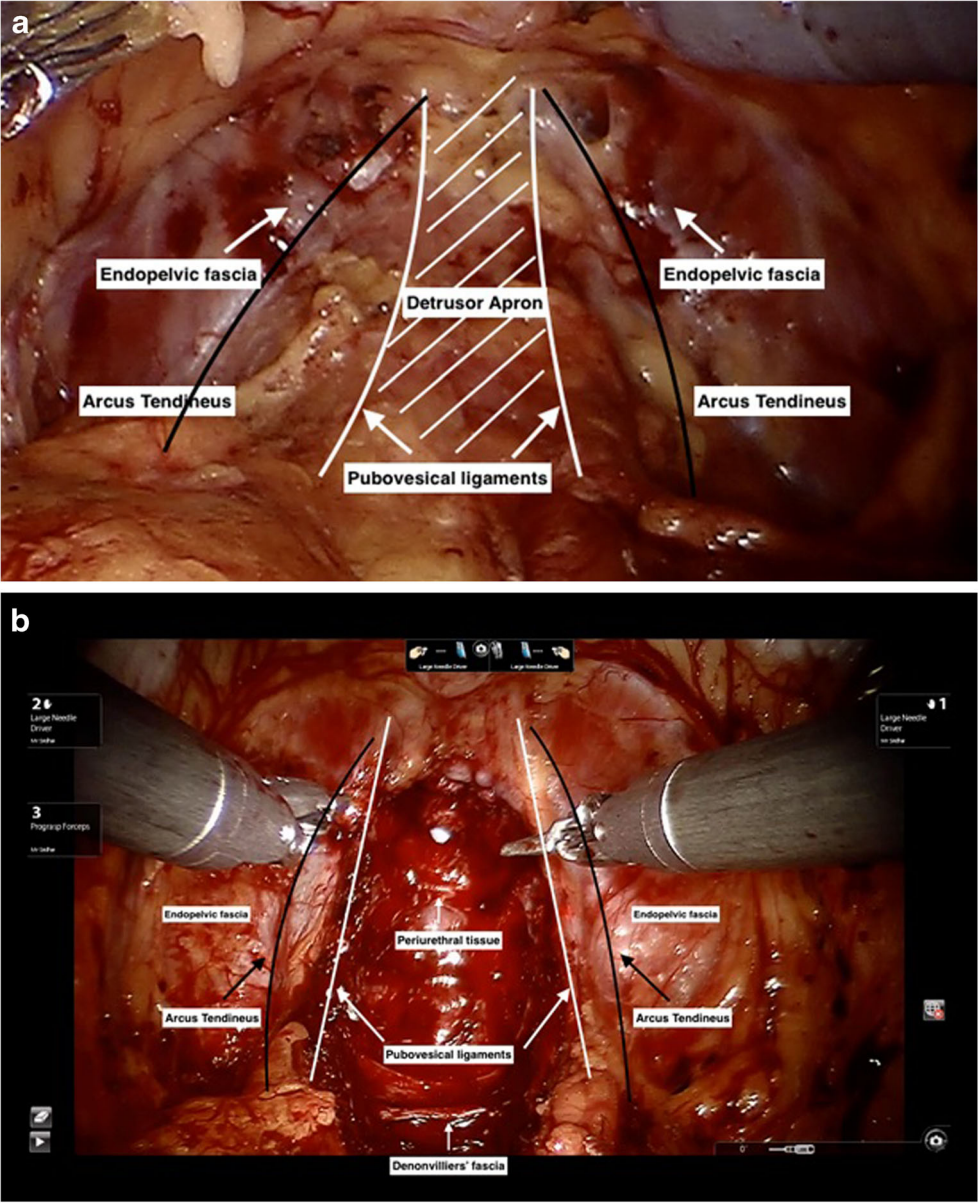
**a** Supporting structures pre-prostate removal. **b** Supporting structures post prostate removal

The urethra lies on a supportive layer that is composed of the puboprostatic collar and pubourethral ligaments anteriorly [12], the arcus tendineus and endopelvic fascia laterally and the Denonvilliers’ fascia running in continuity with the detrusor slips, central perineal tendon, and the levator ani complex posteriorly (akin to the female urethra) Figure 1 [10]. Increased abdominal pressure compresses the urethra against this hammock-like supportive layer, compressing its lumen closed [13]. Fibrosis due to a urethral stricture prevents compression of the urethra by this supportive mechanism. In addition to the pinch-cock effect, they also stabilize the external sphincter. Continuing the incision of the puboprostatic ligament distal to the prostatic apex can weaken the pubourethral ligaments. Similarly, extensive suburethral dissection can also weaken the posterior support.

## Surgical Techniques to Optimize Urinary Continence

These can be broadly divided into techniques that preserve the continence mechanism and those that reconstruct the supportive mechanism.

### Techniques to Preserve the Continence Mechanism

#### Preservation of the Bladder Neck

The bladder neck is composed of three layers of detrusor fibers (the inner longitudinal layer, the middle circular layer, and the outer longitudinal layer) and the internal sphincter. Bladder neck preservation (BNP) aims at preserving these muscle fibers by isolating and dissecting out the prostatic urethra and dividing it as it courses through the prostate. Various approaches to achieve this preservation have been described including an anterior, lateral, or antero-lateral approach [14].

There are limited data, however, on an objective measurement of bladder neck preservation. At our unit, we objectively measure bladder neck size by assessing whether the balloon of a 16 Fr Silicone urethral catheter filled with 1 ml of water can be withdrawn out of the bladder. We consider the bladder neck to be spared if the catheter cannot be withdrawn. This definition has correlated well with return of UC in our series [15].

Nyarangi-Dix et al. [16] performed a randomized clinical trial comparing the UC recovery of 95 men undergoing RARP with and without bladder neck sparing (104 men). The results showed that postoperative UC rates (defined as 1 safety pad or no-pad) at 3, 4, and 12-month were 55.3 versus 84.2% (*p* < 0.001), 74.8 versus 89.5% (*p* = 0.05), and 81.4 versus 94.7% (*p* = 0.02) for patients in the non-BNP versus the BNP group, respectively. The rate of positive surgical margins was not significantly higher in the preservation group. On multivariate analysis, BNP was an independent predictor for UC recovery.

A systematic review and meta-analysis [17] incorporating this and other data from cohort studies demonstrated that BNP improved early UC rates (6 months, OR = 1.66; 95% confidence interval [CI], 1.21–2.27; *p* = 0.001) and long-term urinary UC outcomes (>12 months, OR = 3.99; 95% CI, 1.94–8.21; *p* = 0.0002). This meta-analysis also confirmed that patients who underwent BNP also had lower incidence of bladder neck stricture (OR = 0.49; 95% CI, 0.29–0.81; *p* = 0.006). Anastomotic leak rates, positive surgical margins and biochemical failure rates were not statistically different between the two groups.

Certain factors hinder BNP, such as previous TURP and an enlarged median lobe, and this can have a steep learning curve. Extensive cancer at the base can also make BNP undesirable from an oncologic perspective.

#### Neurovascular Bundle Preservation

Three major sets of nerves are thought to be integral to the functioning of the sphincteric mechanism [11].

- The pudendal nerve, in its classical description, supplies the external striated rhabdosphincter after coursing caudal to the levator ani and away from the field of RARP. However, some authors have suggested the presence of an intrapelvic somatic supply to the rhabdosphincter, located 5.3 ± 1.8 mm [11] from the prostatic apex.
- The internal sphincter has been demonstrated to have dense autonomic nerve supply.
- The cavernosal nerves of the classical neurovascular bundle (NVB) have also been shown to directly innervate the membranous urethra.

These anatomical demonstrations have led some teams to theorize that damage to the NVB might affect the continence mechanism, and preservation does lead to at least earlier UC recovery after RP. This has been physiologically shown by Nelson et al. [18] who measured changes in the intraurethral pressure caused by intraoperative NVB stimulation and noted an increase in pressure on NVB stimulation. Kadono et al. [19] performed urodynamic measurements pre-op, immediately after op, and 1 year after RARP and demonstrated that recovery patterns of storage and voiding functions were the same among non-NVB, unilateral-NVB, and bilateral NVB groups and that higher degrees of NVB preservation contributed to lesser decreases in MUCP and longer functional urethral length (FUL) after RARP.

A systematic review and meta-analysis by Reeves et al. [20] looked at data from 13,749 participants in 27 studies and found that NVB sparing compared with non-NVB sparing resulted in improved early urinary continence rates up to 6 months postoperatively. There was no significant difference beyond this time. This effect was more pronounced for bilateral NVB preservation compared with complete NVB resection. The authors however reported significant variations in baseline characteristics of the compared groups in the individual studies, and also, it was not possible to control for surgical factors such as surgeon experience and differences in surgical technique.

Park et al. [21] interestingly showed that NVB preservation in men with pre-existing erectile dysfunction was associated with improved UC recovery rates. In their study on 360 patients, they found that age (HR: 1.254; 95% CI: 1.002–1.478; *p* = 0.026) and nerve-sparing status (HR: 0.713; 95% CI: 0.548–0.929; *p* = 0.012) were independently associated with recovery of UC on multivariate analysis. This would argue for a direct role of the NVB in the maintenance of UC.

#### Apical Dissection and Preservation of the External Sphincter

The apex of the prostate is in close proximity to the external sphincter and its neural supply. Careful dissection of tissues at the apex of the prostate and avoidance of diathermy, which can result in tissue damage due to dissipation of thermal energy, is commonly believed to improve return of UC. Michl et al. [22] demonstrated this in their retrospective study on 18,427 men that were operated on at a single center. They compared three cohorts of patients. Those who underwent NVB preserving surgery, those who initially underwent NVB preserving surgery, but then had NVB excision due to suggestion of positive margin at the frozen section, and those who underwent wide excision. Their analysis indicated that meticulous apical dissection associated with the NVB preserving radical prostatectomy technique rather than the preservation of the NVBs itself had a positive impact on early and long-term UC rates. They clearly showed that patients who underwent a wide excision surgery had worse UC recovery than those who underwent a meticulous apical dissection (with or without NVB preservation).

The dorsal venous complex (DVC) is a peri-apical structure that is normally divided during the apical dissection. While in open surgery, the DVC is typically ligated prior to its division, and selective suture ligature (SSL) of the DVC has been proposed [23] as a technique to minimize damage to the external sphincter. Lei et al. [24] adapted this technique to RARP and showed that athermal DVC division and subsequent SSL in 303 men had better 5-month UC rates compared to 240 men receiving standard whole ligation and subsequent athermal division, after adjusting for age, BMI, baseline urinary function, and NVB approach.

#### Preservation of Ancillary Anatomical Structures Supporting the External Sphincter

The importance of the anterior support of the urethra by the pubourethral ligaments in continence has been tested in a study measuring the angle of the membranous urethra (AMU) using MRI [25]. Twenty-four patients with post-prostatectomy incontinence were compared to 10 matched patients who were continent after prostatectomy. Patients with incontinence showed significantly wider AMU. Stolzenburg et al. [26] described a puboprostatic ligament preserving technique and showed that this proved to be superior in its clinical outcome for early UC (UC return at 2 weeks 24 vs 12%, *p* = 0.0019; 3 months 76 vs 48%, *p* = 0.0347)

Takenaka et al. [27] have suggested an endopelvic fascia sparing approach, based on an anatomical study in fresh cadavers. Their findings showed that the lower part of the endopelvic fascia covering the levator ani muscle is rich in smooth muscle fibers which interdigitate with the rhabdosphincter. Also, they showed that small nerve branches coming from the pudendal nerve entered the rhabdospincter at 5 and 7 o’clock positions, coursing very closely to levator ani muscle fibers at the level of the apex. They postulated that a conservative approach avoiding the classic incision of the endopelvic fascia would avoid damage to these structures and tested this on 23 consecutive patients. They demonstrated a UC rate of 83, 96, and 100% at 3, 6, and 9 months after radical prostatectomy.

Similarly, Van der Poel et al. [28] measured the impact of a fascial preserving technique (scored subjectively) in a cohort of 151 patients treated with RARP. They deduced that fascial sparing was the best predictor of UC at 6 and 12 months.

Other modifications of the surgical technique to preserve the supportive mechanism include the retzius sparing technique [29] and the pubovesical complex sparing technique [30]. The proponents of these techniques are of the opinion that this maximizes preservation of the periprostatic neural network as well as supportive structures leading to earlier recovery of UC [30].

Lim et al. [29] showed that in a propensity score matched cohort study comparing patients who underwent standard RARP and those who underwent a retzius sparing RARP, UC return at 4 weeks was significantly better in those who underwent retzius sparing surgery (70 vs 50%, *p* = 0.039). Similarly, Galfano et al. [31] and Asimakopoulos et al. [30] have reported almost 90 and 80% immediate UC after catheter removal, respectively. These results were maintained at 1 year. Although these studies are small, they are encouraging and representing an evolution of the learning curve for the achievement of functional outcomes after surgery.

#### Membranous Urethral Length

Preserving a long functional length of the membranous urethra has been demonstrated to regain early UC. Paparel et al. [32]measured the membranous urethral length (MUL) in 64 prostatectomy patients pre- and postoperatively using MRI and showed that patients with a longer pre- and postoperative MUL as well as lesser change in MUL after surgery had earlier return of UC. Similarly, Song et al. [33] in a prospective cohort study of 190 men showed that preoperative MUL ≤16 mm (95% CI 1.01–1.14; *p* = 0.022), postoperative MUL ≤14 mm (95% CI 1.16–9.80; *p* = 0.025), and percent change of MUL >18% (95% CI 1.17–7.23; *p* = 0.021) were significantly associated with urinary incontinence at 6 months. Similar results have been demonstrated in other prospective cohort studies using transrectal ultrasound to measure stretched urethral length and urethral stump length [34].

Schlomm et al. [35] have described their technique for full functional-length urethra (FFLU) preservation during radical prostatectomy. This was achieved by a meticulous apical dissection along anatomic landmarks, preserving the length of the intraprostatically located membranous urethra. By this technique, they demonstrated in 691 men that UC rates were 50.1 and 30.9% 1 week after catheter removal (*p* < 0.0001) for patients with the FFLU technique versus the non-FFLU technique, respectively. In multivariate regression analysis, only the surgical technique correlated significantly with the continence status 1 week after catheter removal. This difference was not demonstrated at 1 year after the surgery. They stressed on the importance of every millimeter (mm) of urethra that could be preserved. Mungovan et al. [36]corroborated this in their systematic review and meta-analysis of pre-operative membranous urethral length and UC recovery and showed that every extra mm of MUL was associated with a faster return to continence (hazard ratio: 1.05; 95% CI: 1.02–1.08, *p* < 0.001; OR: 1.09, 95% CI: 1.05–1.15, *p* < 0.001).

### Reconstructive Techniques to Improve UC Recovery

#### Posterior Reconstruction

Rocco et al. [37] initially suggested a posterior reconstructive technique in open radical prostatectomy for the improvement of UC, and this was adapted to robotic surgery by Coelho et al. [38]. They suggested performing the reconstruction using two 6-in. 3–0 Poliglecaprone sutures tied together. They approximated the free edge of the residual Denonvilliers fascia to the posterior aspect of the rhabdosphincter and the posterior median raphe using one arm of the continuous suture. The second layer of the reconstruction was then performed with the other arm of the suture, approximating the posterior lip of the bladder neck and vesicoprostatic muscle to the posterior urethral edge. They were able to show the benefit of this technique on UC recovery in a RCT and demonstrated that in patients undergoing posterior reconstruction, rates of UC were higher at 1 and 4 weeks after catheter removal (*p* = 0.048 and 0.016, respectively). Continence rates at 12 and 24 weeks were not significantly affected (*p* = 0.908 and *p* = 0.741, respectively). The median time to recovery of UC was also shorter (median: 4 weeks; 95% CI: 3.39–4.61 vs median: 6 weeks; 95% CI: 5.18–6.82; log-rank test, *p* = 0.037).

Since the original description, the posterior reconstruction technique has been tested by other groups with mixed results. A systematic review by Rocco et al. [39] concluded that this was due to the different continence definitions in each analyzed study, several modifications to the original surgical technique, and different surgical approaches. Grasso et al. [40] performed a meta-analysis of available studies and concluded that an overall statistically significant advantage in the rate of postoperative UC in favor of posterior reconstruction at 3–7 days after catheter removal (RR 1.90, 95% CI 1:25–2:90; *p* = 0.003), at 30 days after catheter removal (RR 1.77, 95% CI 1.43–2.20; *p* < 0.001), and at 90 days after catheter removal (RR 1.32, 95% CI 1.10–1.59; *p* = 0.003) was found supporting its incorporation into clinical practice.

#### Combined Anterior and Posterior Reconstruction

Refixation of the puboprostatic ligaments to the anterior aspect of the vesico-urethral anastomosis and reattachment of the tendinous arch to the lateral aspect of the bladder neck can re-instate the anterior supportive structures around the urethra. Very few studies have tested anterior reconstruction in isolation. Tewari et al. [41] in a retrospective cohort study showed that UC return rates were better in those who underwent anterior reconstruction versus standard RARP. It was even better when combined with posterior reconstruction to produce the so-called “total anatomical reconstruction (TAR)” technique.

Two randomized control trials have to date shown improved rates of early UC return after TAR when compared to standard surgery. Hurtes et al. [42] in a multicenter RCT in 74 patients showed that at 1 and 3 months post surgery, the percentage of patients attaining UC (using the University of California Los Angeles Prostate Cancer Index questionnaire) was higher in the reconstructed group when compared to standard RALP (*p* = 0.047 and *p* = 0.016, respectively). Similarly, Student et al. [43], using a pad free definition, showed that in 66 patients randomized to TAR versus standard RALP, UC rate were 21.9 versus 5.9% at 24 h (*p* = 0.079), 43.8 versus 11.8% at 2 weeks (*p* = 0.005), 62.5 versus 14.7% at 4 weeks (*p* < 0.001), 68.8 versus 20.6% at 8 weeks (*p* < 0.001), 75.0 versus 44.1% at 6 months (*p* = 0.013), and 86.66 versus 61.29% at 12 months (*p* = 0.04) after surgery. Similar results have been echoed in other prospective cohort studies [44–47].

#### Vesico-Urethral Anastomosis

A precise approximation of the divided bladder neck and urethra is a crucial step in RARP undertaken through the vesico-urethral anastomosis. Urinary leakage from an incompetent anastomosis can lead to fibrosis and bladder neck stricture [48].

The value of a watertight vesico-urethral anastomosis in postoperative continence was demonstrated in a study comparing interrupted and continuous (47 patients in each group) suturing techniques in retro-pubic radical prostatectomies. The continuous suturing group had a lower rate of leakage (0 vs 10%, *p* = 0.02) and a more rapid return to social continence at 3 months postoperatively (85 vs 66%, *p* = 0.03) [48].

Tuygun et al. [49] performed an MRI scan on 22 patients with post prostatectomy incontinence and a control of 14 patients who are continent after prostatectomy using MRI to detect fibrosis. They found fibrosis around the membranous urethra in all patients with post prostatectomy incontinence, compared to only four continent patients. Paparel et al. [32]showed similar results in their study looking at MRI appearances post surgery and found that patients with a high grade of postoperative periurethral fibrosis tended to have worse postoperative continence.

### Miscellaneous Steps to Improve UC

#### Use of Regenerative Materials

With increased research into the use of regenerative tissue for the treatment of debilitating conditions, it would be natural to assume that this technology could make its way into use for the treatment of urinary incontinence intraoperatively. Patel et al. [50] used a dehydrated human amnion/chorion membrane (dHACM) placed around the NVBs and studies its impact on return of erectile function and UC. In a prospective propensity-matched cohort study in 58 men undergoing RARP, they were able to show that UC at 8 weeks returned in 81.0% of the dHACM group and 74.1% of the no-dHACM group (*p* = 0.373). Mean time to UC was enhanced in the dHACM group (1.21 versus 1.83 months; *p* = 0.033). Other potential substances could include intraoperative injection of stem cells [51] derived from muscle, bone marrow, adipose tissue, or urine around the anastomosis to strengthen the sphincteric mechanism.

#### Intraoperative use of Suburethral Slings

The principle of intraoperative placement of an autologous sling would be to support the membranous urethra and to assist with urethral closure during raised intra-abdominal pressure. Kojima et al. [52] attempted to use an autologous sling made from the vas deferens during RARP and reported that those that had this had a lower IPSS (*p* < .05) and ICIQ-SF (*p* < .05) score 4 weeks after RARP. In addition, mean pad weight gain on 1-h pad test in the sling group was significantly smaller than that in the non-sling group, 4 weeks after RARP (*p* < .05). Valsalva maneuver during cystography demonstrated that the mean posterior urethrovesical angle in the sling group was smaller than that in the non-sling group (*p* < .001).

Cestari et al. [53] also utilized the autologous vas and tested two configurations: standard and 6-branch sling, and they reported that both improved return of UC, and the six-branches suburethral autologous sling was able to increase the rate of early UC recovery compared to the two-arm sling.

No randomized data exist for the use of these slings in clinical practice. The only randomized trial by Nguyen et al. [54] failed to demonstrate a benefit of autologous urethral sling placement at RARP on early return of UC at 6 months 

## Conclusion

Table 1 summarizes the evidence available for various surgical techniques to optimize UC recovery post RARP. High levels of evidence do exist for sparing of the sphincteric mechanism as well as sparing and reconstruction of the supportive structures. Most meta-analyses are limited by the differences in surgical technique, absence of an objective measure of most techniques, and differences in outcome definitions. Unless there is an RCT comparing RARP incorporating all the above steps to RARP without these steps, it is difficult to draw concrete conclusions. In the meantime, a consensus of surgical technique conducted using good qualitative methodology, and experts might suffice to guide surgeons on their learning curve looking to improve the UC outcomes in their patients.

**Table 1:** Surgical techniques to improve UC return post RARP

Surgical technique	Helps UC recovery	No effect	Maximum level of evidence
Bladder neck preservation	*		1a
NVB preservation	*		1b
Meticulous apical dissection	*		2b
Sparing of external sphincter	*		2b
Preservation of supporting structures	*		2b
Maximal preservation of urethral length	*		2b
Posterior reconstruction	*		1a
Total anatomical reconstruction	*		1b
Secure VU anastomosis	*		3
Regenerative materials	*		2b
Autologous slings		*	1b

* implies that that particular step of the procedure has the effect as detailed in the comment heading, number is the accepted level of evidence available
